# A Highly Sensitive D-Shaped PCF-SPR Sensor for Refractive Index and Temperature Detection

**DOI:** 10.3390/s24175582

**Published:** 2024-08-28

**Authors:** Sajid Ullah, Hailiang Chen, Pengxiao Guo, Mingshi Song, Sa Zhang, Linchuan Hu, Shuguang Li

**Affiliations:** State Key Laboratory of Metastable Materials Science & Technology and Key Laboratory for Microstructural Material Physics of Hebei Province, School of Science, Yanshan University, Qinhuangdao 066004, China; sajidktkphy@gmail.com (S.U.); g_pengxiao@163.com (P.G.); songmingshi2023@163.com (M.S.); 18332715635@163.com (S.Z.); linchuan0409@163.com (L.H.)

**Keywords:** D-shaped photonic crystal fiber, surface plasmon resonance, optical fiber sensor, refractive index sensor, temperature sensor

## Abstract

A novel highly sensitive D-shaped photonic crystal fiber-based surface plasmon resonance (PCF-SPR) sensor for dual parameters of refractive index and temperature detecting is proposed. A PCF cladding polishing provides a D-shape design with a gold (Au) film coating for refractive index (RI) sensing (Core 1) and a composite film of silver (Ag) and polydimethylsiloxane (PDMS) for temperature sensing (Core 2). Comsol Multiphysics 5.5 is used to design and simulate the proposed sensor by the finite element method (FEM). The proposed sensor numerically provides results with maximum wavelength sensitivities (WSs) of 51,200 and 56,700 nm/RIU for Core 1 and 2 as RI sensing while amplitude sensitivities are −98.9 and −147.6 RIU^−1^ with spectral resolution of 1.95 × 10^−6^ and 1.76 × 10^−6^ RIU, respectively. Notably, wavelength sensitivity of 17.4 nm/°C is obtained between −20 and −10 °C with resolution of 5.74 × 10^−3^ °C for Core 2 as temperature sensing. This sensor can efficiently work in the analyte and temperature ranges of 1.33–1.43 RI and −20–100 °C. Due to its high sensitivity and wide detection ranges, both in *T* and RI sensing, it is a promising candidate for a variety of applications, including chemical, medical, and environmental detection.

## 1. Introduction

Our world tends toward advancement on daily basis production, standardization of life, living needs and high technology development, which require high sensing technology on an urgent basis [1]. It is necessary to update and produce devices that transmit high amounts of data at high speed and low loss signal processing [2,3]. The role that optical devices play in optical computing, signal processing, and communication networks is considered very crucial at the present time. Optical devices have high signal processing speed, low loss, small size at affordable costs, and immunity to electrical interference compared to other devices [4]. Many sensors are introduced, and surface plasmon resonance (SPR) sensors have similar characteristics that can meet advanced technology requirements. SPR optical sensors possess high sensitivity, real-time monitoring, are label-free, and perform well for many applications in biochemistry, homeland security, medical diagnosis, and food safety. Besides their industrial applications, the advantages of light weight, small size, and simple structure make them one of the most widely used sensors [5,6]. Light incident on a metal-dielectric interface causes electron excitation, and the SPR phenomenon is observed when incident photon frequency matches electron frequency [7]. There are some limitations to SPR sensing in common optical fibers, including over-thick claddings that introduce optical fields. A modification of optical fiber structure is necessary in order to couple guiding light into the cladding and interact with external liquids [8]. In order to overcome these problems, many sensors based on SPR including tapered optical fibers [9], D-shaped optical fiber [10,11], and U-shaped optical fiber [12] are introduced.

Currently, the introduction of photonic crystal fiber (PCF)-based surface plasmon resonance sensors are achieving remarkable progress and widening sensing applications including environmental monitoring, food safety, medical diagnostics and biochemical sensing [6,13,14]. A photonic crystal fiber has the ability to confine light into a hollow core or to confine light in a way that is not possible with traditional fibers [15]. PCF have flexibility in structure, and light can be confined into the air core or be made a solid core by placing air holes surrounding it with the same or different sizes and shapes. PCF-SPR can be coated in two main ways of interior and exterior. Metal coated into air holes is considered interior, while for exterior the metal can be coated on the cladding surface [16]. Variation in the PCF structure is useful for many applications required by industrial needs.

PCF-SPR sensors are greatly dependent on plasmonic metal coatings, where silver, gold, and copper are the most common metals used for their good sensing characteristics [17]. A lot of work has been conducted in the field of refractive sensing, constantly improving with time. In 2020, Yasli et al. presented a PCF-SPR multi-channel sensor and reported maximum wavelength sensitivity (WS) of 3083 nm/RIU using the finite element method [18]. Jain et al. proposed a PCF-SPR sensor for a wide range of RI-sensing applications and reported WS and amplitude sensitivity (AS) of 10,000 nm/RIU and 1115 RIU^−1^ along with a sensor resolution of 2 × 10^−5^ RIU [15]. The authors realized that the sensor operates in the wavelength and the RI ranges of 500 to 1350 nm and 1.35 to 1.40. Despite its positive results in many cases, it still requires improvement to overcome the complexity of its structure and sensing system instability. Further, some research indicates that special types of PCF can enhance sample detection, including U-shaped and D-shaped PCFs [19,20]. The introduction of special PCF and symmetry breaking aims to excite its birefringence effect. Additionally, it is possible to improve sensitivity by depositing new materials on metal surfaces, such as indium tin oxide, MoS2, and graphene [21,22]. On the other hand, temperature plays a very important role in biological activity materials and its control is very important for smooth process. PCF sensor obtains the richest features after the introduction of naturally temperature-sensitive materials into PCF. Luan et al. proposed a PCF-based temperature sensor by filling chloroform and ethanol in air holes and reported temperature sensitivity of 4 nm/°C between −4 and 15 °C [23]. Yang et al. presented a PCF-SPR sensor with embedded silver wires for wide temperature detection ranges of about 20–320 °C and reported a maximum temperature sensitivity of 5.0 nm/°C and a temperature resolution of 0.0200 °C [24]. The measurements of refractive index (RI) and temperature (T) are closely related, so the development of dual-parameter sensors that can simultaneously measure both the RI and T is of great importance for practical applications. Several reports have described dual-parameter PCF-SPR sensors that simultaneously measure the RI and T. Guo et al. presented a D-shaped PCF amphibious sensor for RI and T sensing with liquid crystals. The authors reported a high sensitivity of 2275 nm/RIU along with an amplitude sensitivity of 88.2 RIU^−1^ for 1.0 to 1.6 RI, and also maximum temperature and amplitude sensitivity were −0.311 °C and 9.09 nm/°C in the range of 15 to 50 °C [25]. Gao et al. experimentally demonstrated high sensitivity and a wide sensing range optical fiber sensor by splicing MMF-PCF-MMF. They showed RI sensitivity of 3341.65 nm/RIU between 1.333 and 1.3953 and T sensitivity of 2.02 nm/°C between −20 and 100 °C [26]. Yin et al. theoretically studied a dual-channel PCF-SPR sensor for RI and T sensing where sodium was used as a plasmonic material. They reported a maximum RI sensitivity of 8700 nm/RIU in the wavelength range of 600–1700 nm when the analyte changed from 1.33 to 1.41 RI and a maximum T sensitivity of 20.20 nm/°C in the range of 0–50 °C [27].

Based on the advantages and features of a special PCF-SPR sensor over a traditional optical sensor, we propose a D-shaped PCF-SPR sensor. The sensor is proposed for dual-parameter sensing using gold deposited on a polished surface at Core 1 for refractive index sensing and silver and PDMS at Core 2 for temperature sensing. The numerical results we obtain show high RI sensitivities of 51,200 and 56,700 nm/RIU along with resolutions of 1.95 × 10^−6^ and 1.76 × 10^−6^ RIU for Core 1 and Core 2 using gold and silver, respectively, with the analyte range of 1.33–1.43 RI. Also, by adding polydimethylsiloxane (PDMS) to the silver metal surface, we observe high temperature sensitivity of 17.4 nm/°C between −20 and −10 °C for Core 2. There are very few sensors that have good performance for both RI and T parameters, and to our knowledge, our proposed sensor has both high RI and T sensitivity. In addition to its good performance, our proposed sensor has a simple structure and is easy to prepare. This sensor is designed to work in food safety, industrial production, environmental monitoring, and bio- and chemical-related RI and T sensing.

## 2. Design of Dual-Parameter PCF-SPR Sensor

**Figure 1:**
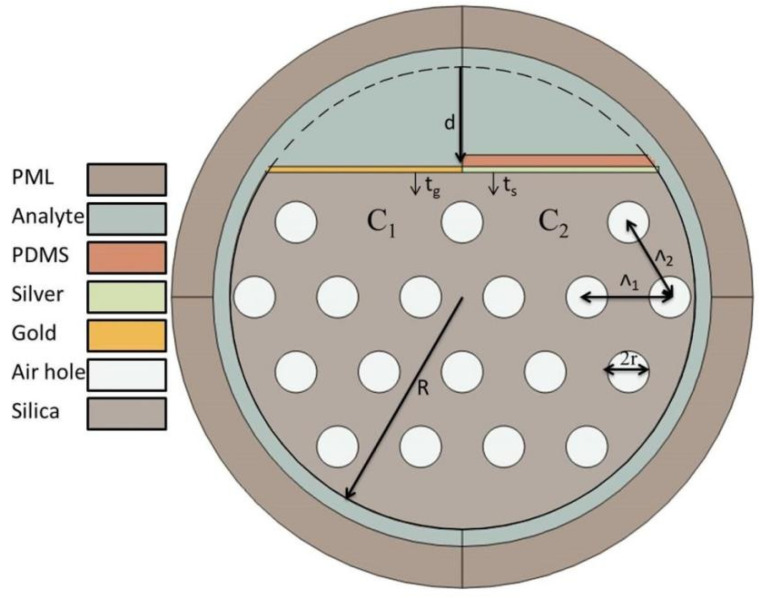
A D-Shaped PCF with two cores, gold film, and composite film of silver + PDMS.

Designing an appropriate and simple structure which can deal with the existing technology is very challenging for optical fiber device applications. There are a lot of things that need to be kept in mind in order to meet the advanced requirements. Our previous work, based on a dual-sided polished PCF with a simple design, showed tremendous improvement in wavelength sensitivity [28]. Based on the previous work, we propose a D-shaped PCF-SPR sensor in Figure 1 by taking related matters into consideration. Variation in structural parameters is always effective in appropriate design. The proposed structure contains a total of 18 air holes of the same size adjusted in a proper way to avoid confusion, while silica material is used in the cladding. Gold is deposited on the polished surface to produce Core 1 for refractive index sensing, and silver + PDMS is deposited for Core 2 to achieve temperature sensing. Structural parameters include metal thicknesses in a range from 40 to 60 nm and polishing depths from 12 to 14 µm. Temperature and analyte RI is also varied for Core 1 and Core 2, respectively, to determine the effect and independence of dual channels on each other. Table 1 provides optimal parameters for the proposed structure.

The cladding of the proposed sensor is made of silica material, and the RI can be calculated using a well-known Sellmeier formula as follows [29]:

(1) $$n^{2}=1+\frac{A_{1}\lambda^{2}}{\lambda^{2}-B_{1}}+\frac{A_{2}\lambda^{2}}{\lambda^{2}-B_{2}}+\frac{A_{3}\lambda^{2}}{\lambda^{2}-B_{3}}$$

In the above formula, *n* is used for the RI and dependent on wavelength *λ*, where *λ* is the incident light wavelength. *A*_1_, *A*_2_, and *A*_3_ have values of 0.6961663, 0.4079426, and 0.8974794, while *B*_1_, *B*_2_, and *B*_3_ with values of 0.068404, 0.1162414, and 9.896161 µm are constants in the above formula. We use two metals as plasmonic materials, and their dielectric constants can be calculated using the Drude–Lorentz model formula as follows:

(2) $$\varepsilon_{m}=\varepsilon_{\infty }-\frac{\omega_{D}^{2}}{\omega \left(\omega +j\gamma_{D}\right)}-\frac{\Delta \varepsilon .\Omega_{L}^{2}}{\left(\omega^{2}-\Omega_{L}^{2}\right)+j\Gamma_{L}\omega }$$

where the metal dielectric constant is denoted by *ε_m_*, *ε_∞_* indicating the dielectric constant for high frequency and the weighting factor with Δ*ε.* Terms *ω_D_*, *γ_D_*, and *ω* are used for the plasma, damping, and angular frequencies, while Ω*_L_* and *Γ_L_* are used for Lorentz oscillator frequency and spectral width. Table 2 lists the values of this equation.

**Figure 2:**
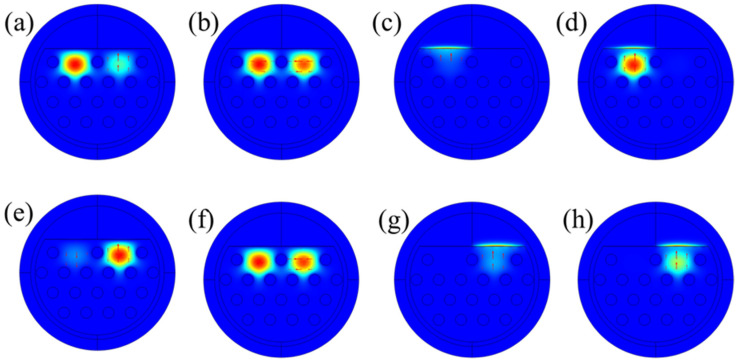
Electric field distribution for Core 1. (**a**) Y-P and (**b**) X-P core modes at wavelengths of 620 nm. (**c**) Y-P cladding defect mode, and (**d**) Y-P super core defect mode at SPR with wavelengths of 1135 nm. RI = 1.42. Electric field distribution for Core 2. (**e**) Y-P and (**f**) X-P core modes at wavelengths of 720 nm. (**g**) Y-P cladding defect mode, and (**h**) Y-P super core defect mode at SPR with wavelength of 1291 nm. T = −20 °C.

Electric field distribution for Core 1 and 2 with analyte RI = 1.42 and T = 30 °C, respectively, are shown in Figure 2. Figure 2a and Figure 2e show Y-polarization (Y-P) while Figure 2b and Figure 2f show X-polarization (X-P) core modes for Core 1 and 2, respectively, at wavelengths of 620 and 720 nm. At lower wavelengths, most of the light is transmitted in the core, while at higher wavelengths, and especially at resonance wavelengths of the corresponding peak, the core light is leaked towards the PCF cladding. Cladding and core defect modes for Core 1 at a resonance wavelength of 1135 nm is shown in Figure 2c and Figure 2d, where the maximum possible light is leaked from core to cladding and the SPR phenomenon is obtained. Similarly, Figure 2g and Figure 2h show cladding and core defect modes for Core 2 at a 1291 nm resonance wavelength.

The most widely used temperature sensing material is Polydimethylsiolxane (PDMS) coating on metal surfaces. It can work as a protective layer to avoid oxidation of Ag metal as well as change the resonance wavelength with temperature variations. The temperature and RI relationship for PDMS can be calculated as follows [32]:

(3) $$n_{PDMS}=-4.5\times 10^{-4}\cdot T+1.4176$$

PDMS values corresponding to temperature are listed in Table 3.

## 3. Sensor Model Manufacturing Procedure

Different techniques provide PCF structure; the most common are stack-and-stretch and extrusion. Russel et al. [33] manufactured PCF using the mentioned techniques. Implementing the above techniques requires an initial PCF structure. In our previous work [28], we showed steps for constructing the initial PCF structure, and similar steps are used here as well.

**Figure 3:**
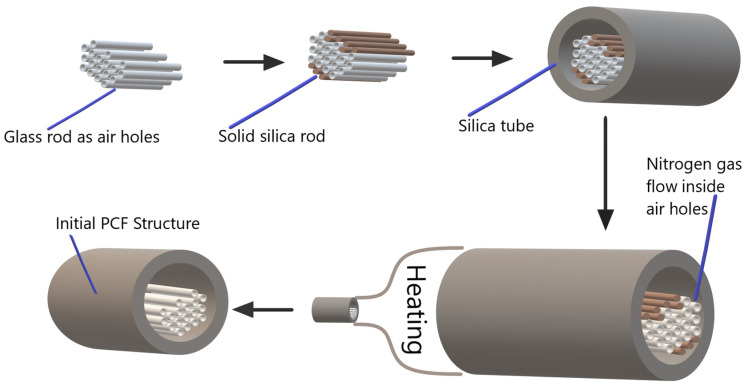
Schematic of PCF structural manufacturing.

Figure 3 shows the manufacturing of the initial PCF structure where we need to take hollow tubes that are slightly bigger than the original PCF structure. Our first step is the proper arrangement of our proposed sensor’s air holes using glass rods. To fill the hollow silica tube, a solid silica rod is used in the second step. Glass and solid silica rods are placed inside the hollow silica tube as the third step. As a next step, a heating process of 1000 to 1700 °C is carried out with continuous nitrogen gas flow inside air holes to prevent blockages. During the heating process, hollow and solid silica melt, creating an initial PCF structure with air holes. The initial PCF structure is polished further to achieve a D-shaped structure, and the metal layers are coated.

## 4. Results and Discussion

### 4.1. Dispersion Relation

The proposed sensor is designed and simulated using finite element method based on Comsol Multiphysics 5.5 software. For light absorption, a perfectly matched layer is used. A PCF-SPR sensor relies heavily on incident light interaction with plasmonic material surface electrons. Surface plasmon wave (SPW) is generated when incident electrons strike with free electrons and phase matches when resonance condition occurs. At resonance condition, the effective refractive index of the core is equal to the plasmonic mode at a specific wavelength, enabling strong light couples, resulting in maximum energy transfer from core to the plasmonic mode. A sharp loss peak is obtained that can be changed quickly with small changes in the analyte RI. An important parameter for sensor performance evaluation is confinement loss, and it is calculated as follows:

(4) $$L=8.686\times \frac{2\pi }{\lambda }Im\left(n_{eff}\right)\times 10^{4}\;dB/m$$

where *I*m (*n_eff_*) is the imaginary part of the effective RI and *λ* is the working wavelength of the incident wavelength at the nanoscale.

**Figure 4:**
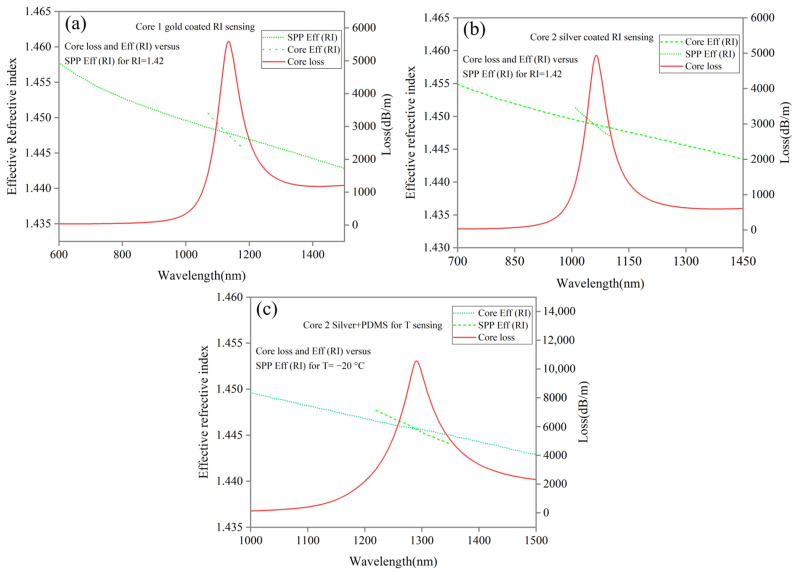
Dispersion relation between the core and SPP modes. SPR Phenomenon (**a**) Core 1 with gold coated at wavelength of 1135 nm for RI sensing, (**b**) Core 2 with silver coated at wavelength of 1064 nm for RI sensing, and (**c**) Core 2 with silver + PDMS coated at wavelength of 1291 nm for temperature sensing.

A dispersion relationship and a phase matching condition are shown in Figure 4a–c for Core 1 and 2 coated with gold and silver, respectively, as RI sensing for Y-polarization and temperature sensing for Core 2 when coated with silver + PDMS. As RI sensing, SPR occurs for Core 1 and 2 for analyte RI =1.42 at resonance wavelengths of 1135 and 1064 nm, respectively, shown in Figure 4a,b. As a temperature sensor, Figure 4c shows SPR for Core 2 coated with silver + PDMS at a resonance wavelength of 1291 nm for T = −20 °C. As shown above, phase matching is for the first-order SPP with core modes and confinement loss of the core mode reaching its maximum value during coupling.

### 4.2. Confinement Loss Spectra

**Figure 5:**
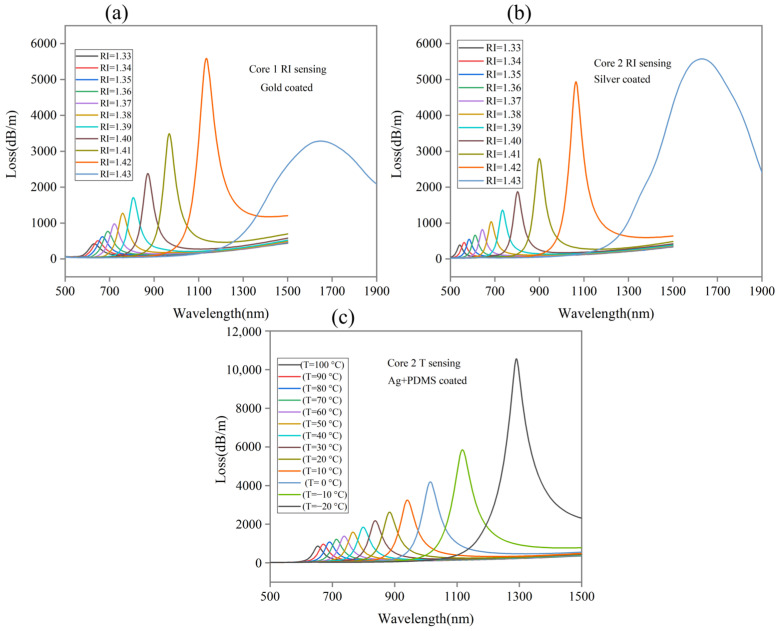
Core loss vs. wavelength for different analyte RI (**a**) gold with Core 1 and (**b**) silver with Core 2. (**c**) Core loss vs. wavelength with various temperatures for silver + PDMS at Core 2.

The confinement loss (CL) spectra for Core 1 as RI and Core 2 as RI and temperature sensing are presented in this part. Figure 5a shows CL vs. wavelength ratio for Core 1 with gold coated in the analyte ranging from 1.33 to 1.43 RI. Using a wavelength gap of 1 nm, the proposed sensor performance is analyzed. The results show that the resonance wavelengths are 631, 648, 668, 692, 722, 759, 807, 872, 968, 1135, and 1647 nm for the corresponding analyte RI of 1.33, 1.34, 1.35, 1.36, 1.37, 1.38, 1.39, 1.40, 1.41, 1.42, and 1.43, respectively, when T = 30 °C. The highest loss peak of 5589.5 dB/m is obtained for analyte RI = 1.42.

Similarly, Figure 5b illustrates the performance of Core 2 with silver coated in the analyte ranging from 1.33 to 1.43 RI and the resonance wavelengths are 542, 562, 584, 611, 643, 684, 734, 802, 900, 1064, and 1631 nm for the corresponding analyte RI of 1.33, 1.34, 1.35, 1.36, 1.37, 1.38, 1.39, 1.40, 1.41, 1.42, and 1.43, respectively, when T = 30 °C. The highest loss peak of 5575.3 dB/m is obtained for analyte RI = 1.43. Figure 5a,b shows RI sensing, and the obtained results show that the lowest resonance wavelengths peak at low RI of 1.33 while the highest resonance wavelengths peak at high RI of 1.43. An increase in analyte RI results in an increase in corresponding peak resonance wavelengths. Such an increment is suitable for RI sensing and referred to as red-shifted.

Figure 5c shows CL vs. a wavelength ratio for Core 2 with silver + PDMS coated in temperatures ranging from −20 to 100 °C. The results show that the resonance wavelengths are 1291, 1117, 1014, 940, 884, 837, 799, 766, 738, 713, 691, 671, and 653 nm for the corresponding temperatures of −20, −10, 0, 10, 20, 30, 40, 50, 60, 70, 80, 90, and 100 °C. Figure 5c shows that the highest resonance wavelength peak occurs at T = −20 °C, while the lowest resonance wavelength peak occurs at T = 100 °C. The highest loss peak of 10,561 dB/m is obtained at a temperature of −20 °C. With increasing temperature, the corresponding resonance wavelength peaks are moving towards shorter wavelengths. As a result, it clearly indicates that the data are blue-shifted, which is considered to be better for temperature sensing.

### 4.3. Dual-Channel Independence

**Figure 6:**
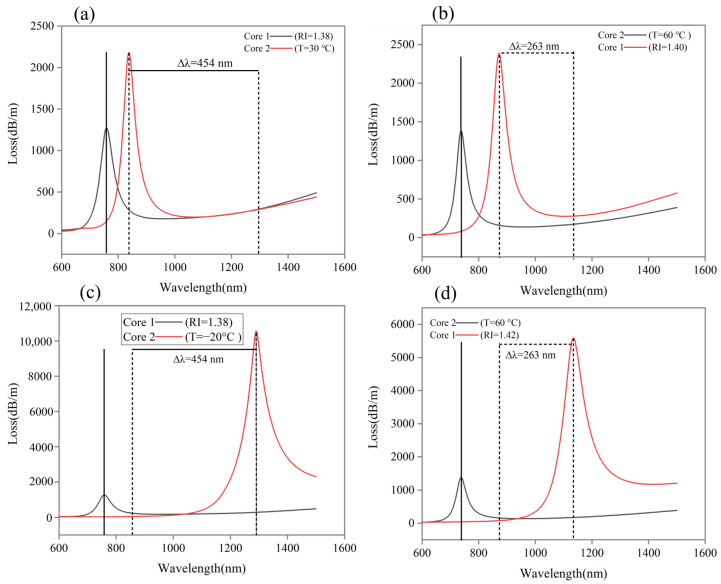
Loss spectrum (**a**,**c**) when RI = 1.38 is constant for Core 1 and *T* changes from 30 to −20 °C for Core 2. (**b**,**d**) When *RI* changes from 1.40 to 1.42 for Core 1 and *T* = 60 °C is constant for Core 2.

Our proposed sensor is for dual-parameter sensing which measures RI and T simultaneously. To ensure that dual sensing works properly, the sensors must be independent of one another and their performance should not be affected by one another. As shown in Figure 6a,b, we keep RI =1.38 of Core 1 constant while changing temperature from 30 to −20 °C of Core 2. The obtained results provide a wavelength shift of 454 nm for Core 2, while Core 1 does not change. Similarly, in Figure 6c,d, we keep a constant temperature of 60 °C for Core 2 and change RI from 1.40 to 1.42 for Core 1. The corresponding resonance peak of Core 1 moves about 263 nm, while Core 2 remains unchanged. The proposed sensor exhibits excellent performance in dual-channel independence, and the channels do not affect each other’s performance, which is ideal for dual-parameter sensing.

### 4.4. Sensitivity-Based Performance

**Figure 7:**
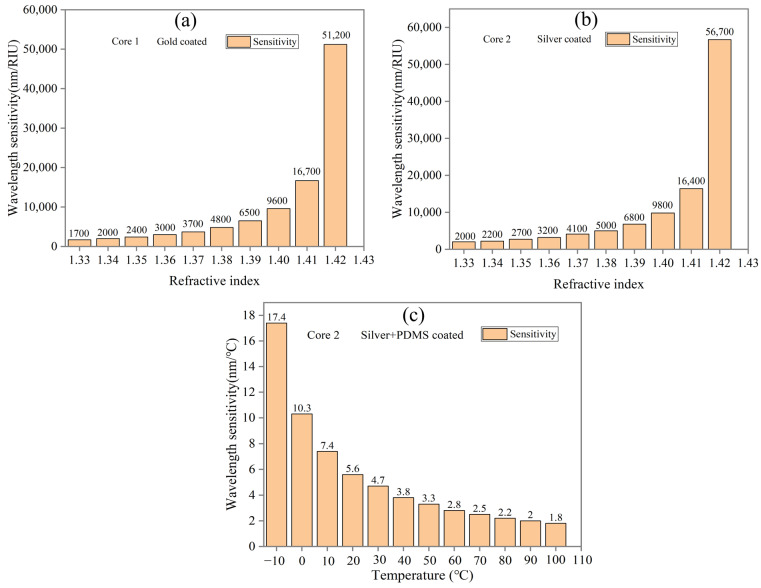
Wavelength sensitivity of (**a**) Core 1 with gold coat, (**b**) Core 2 with silver coat for refractive index, and (**c**) Core 2 with silver + PDMS coat for temperature.

Sensor sensitivity is the basic parameter that determines the performance of an SPR sensor. Wavelength sensitivity (WS) of RI is a measure of resonance wavelength difference of the corresponding peak caused by changing RI of the analyte. In fact, if a small variation in the analyte RI provides high-resonance wavelength shifts, it indicates high sensor sensitivity. The wavelength interrogation method is used for wavelength sensitivity calculation and is expressed as follows:

(5) $$S_{w}(\lambda )=\frac{∆\lambda_{peak}}{∆n_{a}}$$

where $S_{w}(\lambda )$ refers to wavelength sensitivity, $∆\lambda_{peak}$ is resonance wavelength difference, and $∆n_{a}$ is used for analyte RI changes. By applying the WS equation, we calculate the proposed sensor sensitivity. Figure 7a shows that at lower RI, wavelength shift is small, and with increasing RI, wavelength shift increases. Figure 7a shows that the highest sensitivity of 51,200 nm/RIU for Core 1 is obtained between 1.42 and 1.43 RI. Similarly, if we consider Core 2 as RI sensing and calculate sensitivity using the WS equation, we obtain a high sensitivity of 56,700 nm/RIU between 1.42 and 1.43 RI as shown in Figure 7b. The detailed performance for Cores 1 and 2 as RI sensing is shown in Table 4 and Table 5.

Temperature sensitivity (TS) of the proposed sensor is calculated by replacing ∆*n_a_* with ∆T in the above Equation (5). The temperature sensitivity of the proposed sensor for Core 2 is shown in Figure 7c. According to Figure 7c, resonance wavelength shifts are greater at low temperatures and decrease at higher temperatures. The lowest TS of 1.8 nm/°C is obtained between the temperatures of 90 and 100 °C, while the highest TS of 17.4 nm/°C is obtained between −20 and −10 °C. Based on the figure results, TS decreases with increasing temperature, and the proposed sensor’s average TS is about 5.32 nm/°C. Table 6 shows the detailed performance of Core 2 as a temperature sensor.

**Figure 8:**
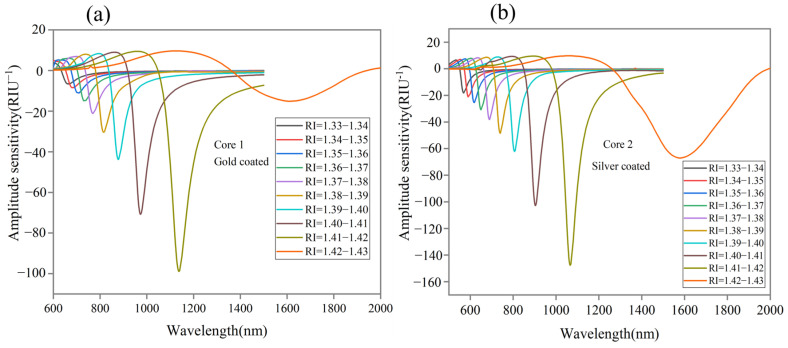
Amplitude sensitivity of (**a**) Core 1 with gold coat and (**b**) Core 2 with silver coat.

Another significant factor on which sensor performance can be judged as amplitude sensitivity (AS). A formula is used for calculating AS as follows:

(6) $$S_{A}\left(\lambda \right)=-\frac{1}{\alpha \left(\lambda ,n_{a}\right)}\frac{\delta \alpha \left(\lambda ,n_{a}\right)}{\delta n_{a}}$$

where the term $S_{A}\left(\lambda \right)$ indicates AS, $\alpha \left(\lambda ,n_{a}\right)$ is loss of specific RI, $\delta \alpha \left(\lambda ,n_{a}\right)$ is the loss difference of adjacent RI, and $\delta n_{a}$ refers to variation in the analyte RI. The proposed sensor is simulated ranging from 1.33 to 1.43 RI, and the maximum AS of −98.9 RIU^−1^ is obtained for Core 1 at a wavelength of 1138 nm for 1.41–1.42 RI as shown in Figure 8a. Also, Figure 8b shows an AS of −147.6 RIU−1 for Core 2 at the wavelength of 1066 nm for 1.41–1.42 RI.

### 4.5. Sensor Resolution

Sensor resolution is the capability of measuring the smallest variations in the RI or temperature. Sensor resolution can be measured by the following formula:

(7) $$R\left(RI\right)=∆z\frac{\Delta \lambda_{min}}{\Delta \lambda_{peak}}$$

where *R* is used for resolution, ∆*z* refers to changes in *RI* or *T*, $\Delta \lambda_{min}$ is the minimum resolution of spectrometer, and $\Delta \lambda_{peak}$ indicates loss peak resonance wavelength difference of the adjacent *T* or *RI*. In this formula, we use $\Delta \lambda_{min}$ = 0.1 nm as spectrometer minimum resolution which is similar to the actual resolution of the spectrometer. ∆*z* = 0.01 for *RI* and ∆*z* = 10 °C for temperature resolution. By using the above formula, we obtain resolutions of 1.95 × 10^−6^ and 1.76 × 10^−6^ RIU for Core 1 and 2, *RI* sensing, and a resolution of 5.74 × 10^−3^ °C for Core 2, temperature sensing. The detailed performance of *RI* sensing resolutions are shown in Table 4 and Table 5, while the temperature sensor resolutions are shown in Table 6.

### 4.6. Structural Parameter Variation

**Figure 9:**
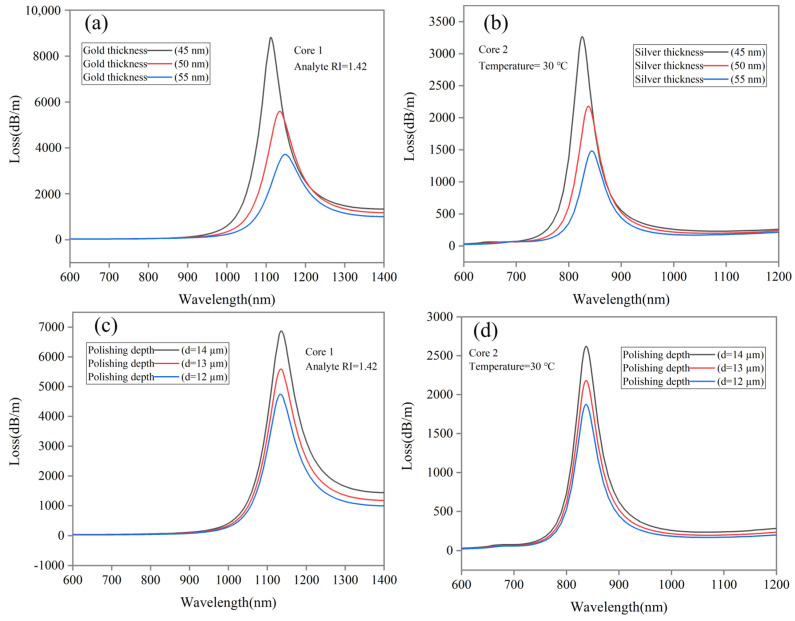
Variation of structural parameters. (**a**,**c**) Gold and silver metal thicknesses (45, 50 and 55 nm). (**b**,**d**) Polishing depth (12, 13 and 14 µm) for Core 1 at RI = 1.42 and Core 2 at T = 30 °C.

This section illustrates the sensor performance by varying structural parameters. Figure 9a,c shows how Core 1 with 1.42 RI and Core 2 with 30 °C respond when changing metal thickness between 45 and 55 nm. Core 1 and 2 resonance wavelengths for the corresponding peaks shift towards the longer wavelength by increasing the thickness of the metal, although somewhere the loss peak decreases, but it is red-shifted by increasing thickness. The proposed sensor is designed with a 50 nm thickness that works for a wide detection range with sharp peaks. Additionally, we polish the D shape differently and test the performance of the design at polishing depths of 12–14 µm shown in Figure 9b,d. According to simulation results, as polishing depth increases, the corresponding peak shifts to longer wavelengths. The obtained results are in accordance with the variation in structural parameters, consistent with our previous results and with the actual performance of PCF sensor. The comparison of different sensors with our proposed sensor is shown in Table 7. Our proposed sensor has better performance than those reported in the literature.

## 5. Conclusions

We present a highly sensitive dual-parameter D-shaped PCF-SPR sensor for simultaneous measurements of RI and temperature in this paper. Gold, being the most stable material, is deposited for Core 1 to ensure prolonged operation as a refractive index (RI) sensor, while PDMS is deposited on a silver surface for Core 2, suitably working as a temperature sensor. The proposed sensor is designed to detect analytes and temperatures outside, both of which can easily be varied. Our current research shows improvements in both detection ranges and sensitivities. By using optimum parameters and the proposed design, we obtain maximum wavelength sensitivities (WSs) of 51,200 and 56,700 nm/RIU between 1.42 and 1.43 RI along with a spectral resolution of 1.95 × 10^−6^ and 1.76 × 10^−6^ RIU, respectively, for Core 1 and 2 as RI sensing. Amplitude sensitivities for Core 1 and 2 are −98.9 and −147.6 RIU^−1^. Also, for Core 2, the obtained results show a maximum WS of 17.4 nm/°C between −20 and −10 °C along with a resolution of 5.74 × 10^−3^ °C as temperature sensing. Based on the comparison with many reported sensors, our proposed sensor possesses high sensitivity, high resolution, low price, and a wide detection range. This sensor design can efficiently work for simultaneously measuring RI and T, and has a variety of applications in the field of biosensing and environmental and chemical sensing.

## Figures and Tables

**Table 1:** Parameters of the proposed sensor.

Cladding radius R = 28 µm	Air hole radius r = 2.5 µm	Pitch 1 ᴧ_1_ = 10 µm	Pitch 2 ᴧ_2_ = 12 µm	Gold thickness t_g_ = 50 nm
Silver thickness t_s_ = 50 nm	Polishing depth d = 13 µm	Analyte layer a = 2 µm	Perfectly matched layer PML = 5 µm	

**Table 2:** Optimized values for silver (Ag) and gold (Au).

	Physical Significance	Dielectric Constant Frequency	Plasma Frequency	Weighting Factor	Frequency of Lorentz Oscillator	Damping Frequency	Spectral Width	Reference
Metal	Constant	ε_∞_	ωD/2π (THz)	Δε	Ω*_L_*/2π (THz)	γ_D_/2π (THz)	*Γ_L_*/2π	
Ag		2.4064	2214.6	1.6604	1330.1	4.8	620.7	[30]
Au		5.9673	2113.6	1.09	650.07	15.92	104.86	[31]

**Table 3:** n_PDMS_ values corresponding to temperature.

Temp (°C) =	−20	−10	0	10	20	30	40	50	60
n_PDMS_ =	1.4266	1.4221	1.4176	1.4131	1.4086	1.4041	1.3996	1.8951	1.3906
Temp (°C) =	70	80	90	100					
n_PDMS_ =	1.3861	1.3816	1.3771	1.3726					

**Table 4:** Performance as RI sensing for Core 1.

RI	Loss (dB/m)	Resonance Wavelength (nm)	Analyte (RI) Difference	Resonance Wavelength SHIFT (nm)	Refractive Index SENSITIVITY (nm/RIU)	Resolution (RIU)
1.33	422.16	631	1.34–1.33	17	1700	5.88 × 10^−5^
1.34	506.36	648	1.35–1.34	20	2000	5.00 × 10^−5^
1.35	617.97	668	1.36–1.35	24	2400	4.16 × 10^−5^
1.36	768.62	692	1.37–1.36	30	3000	3.33 × 10^−5^
1.37	976.79	722	1.38–1.37	37	3700	2.70 × 10^−5^
1.38	1272.7	759	1.39–1.38	48	4800	2.08 × 10^−5^
1.39	1708	807	1.40–1.39	65	6500	1.54 × 10^−5^
1.40	2380	872	1.41–1.40	96	9600	1.04 × 10^−5^
1.41	3488.9	968	1.42–1.41	167	16,700	5.99 × 10^−6^
1.42	5589.5	1135	1.43–1.42	512	51,200	1.95 × 10^−6^
1.43	3283.7	1647				

**Table 5:** Performance as RI sensing for Core 2.

RI	Loss (dB/m)	Resonance Wavelength (nm)	Analyte (RI) Difference	Resonance Wavelength Shift (nm)	Refractive Index Sensitivity (nm/RIU)	Resolution (RIU)
1.33	387.62	542	1.34–1.33	20	2000	5.00 × 10^−5^
1.34	457.36	562	1.35–1.34	22	2200	4.54 × 10^−5^
1.35	546.06	584	1.36–1.35	27	2700	3.70 × 10^−5^
1.36	662.26	611	1.37–1.36	32	3200	3.12 × 10^−5^
1.37	817.79	643	1.38–1.37	41	4100	2.44 × 10^−5^
1.38	1036.7	684	1.39–1.38	50	5000	2.00 × 10^−5^
1.39	1358	734	1.40–1.39	68	6800	1.47 × 10^−5^
1.40	1873.7	802	1.41–1.40	98	9800	1.02 × 10^−5^
1.41	2795	900	1.42–1.41	164	16,400	6.09 × 10^−6^
1.42	4936.4	1064	1.43–1.42	567	56,700	1.76 × 10^−6^
1.43	5575.3	1631				

**Table 6:** Performance of Core 2 as a temperature sensor.

PDMS (RI)	Loss (dB/m)	Resonance Wavelength (nm)	Temperature (°C)	Resonance Wavelength Shift (nm)	Temperature Sensitivity (nm/°C)	Resolution (RIU)
1.3726	868.19	653	100	18	1.8	5.55 × 10^−2^
1.3771	965.61	671	90	20	2.0	5.00 × 10^−2^
1.3816	1080.1	691	80	22	2.2	4.54 × 10^−2^
1.3861	1217.2	713	70	25	2.5	4.00 × 10^−2^
1.3906	1382.1	738	60	28	2.8	3.57 × 10^−2^
1.3951	1588	766	50	33	3.3	3.03 × 10^−2^
1.3996	1847.6	799	40	38	3.8	2.63 × 10^−2^
1.4041	2179.8	837	30	47	4.7	2.12 × 10^−2^
1.4086	2624.3	884	20	56	5.6	1.79 × 10^−2^
1.4131	3246.2	940	10	74	7.4	1.35 × 10^−2^
1.4176	4191.7	1014	0	103	10.3	9.70 × 10^−3^
1.4221	5849	1117	−10	174	17.4	5.74 × 10^−3^
1.4266	10561	1291	−20			

**Table 7:** Comparison of the proposed sensor with those in the literature.

Type of Sensors	WL Range RI (nm)	Range in Term of (RI)	W.S (nm/RIU)	A.S (RIU^−1^)	Resolution (RIU)	Ref.
	T (nm)	T	(nm/°C)	(°C^−1^)		
High-sensitivity SPR-PCF sensor for RI and T	1.43–1.50	36–86	44,850	NA	NA	[1]
			16.875			
Ultra-high-sensitivity D-shaped PCF RI sensing	NA	1.33–1.395	18,612	NA	4.16 × 10^−6^	[11]
		20–60	2.0			
PCF-SPR for wide range of RI sensing	500–1350	1.35–1.40	10,000	1115	2 × 10^−5^	[15]
Dual-Core PCF-SPR for RI sensing	576–896	1.33–1.42	28,000	6829	3.57 ×10^−6^	[16]
Amphibious T and RI sensor with a D shape	2097–1078	1.0–1.6	2275	−88.2		[25]
		15–50	9.09	−0.311		
Broadband dual-channel sensor	600–1050	1.33–1.41	8700	NA	2.32 × 10^−5^	[27]
	1051–1700	0–50	20.2		9.36 × 10^−3^	
H-Shaped Dual-Channel SPR sensor	NA	1.33–1.36/1.37–1.40	4000/10,500	484/288	2.5 × 10^−5^/9.09 × 10^−6^	[34]
Dual-Core PCF-SPR for RI	450–1150	1.33–1.44	11,200	505.037	8.92 × 10^−6^	[31]
Our previous work of highly sensitive PCF-SPR sensor for RI sensing	632–1269	1.33–1.42	27,400	100.43	3.64 × 10^−6^	[28]
Dual-Core PCF-SPR for RI and T sensing	542–1647	1.33–1.43	51,200/56,700	98.9/147.6	1.95 × 10^−6^/1.76 × 10^−6^	This work
	653–1291	−20–100	17.4	NA	5.74 × 10^−3^	

## Data Availability

The data that support the findings of this study are available upon reasonable request from the authors.
